# Role of Gut Microbiota Dysbiosis in Breast Cancer and Novel Approaches in Prevention, Diagnosis, and Treatment

**DOI:** 10.7759/cureus.17472

**Published:** 2021-08-26

**Authors:** Sheila W Ruo, Tasnim Alkayyali, Myat Win, Anjli Tara, Christine Joseph, Amudhan Kannan, Kosha Srivastava, Olive Ochuba, Jasmine K Sandhu, Terry R Went, Waleed Sultan, Ketan Kantamaneni, Sujan Poudel

**Affiliations:** 1 General Surgery, California Institute of Behavioral Neurosciences & Psychology, Fairfield, USA; 2 Internal Medicine, Marmara University, Istanbul, TUR; 3 Pathology, California Institute of Behavioral Neurosciences & Psychology, Fairfield, USA; 4 General Surgery, Nottingham University Hospitals NHS Trust, Nottingham, GBR; 5 General Surgery, Liaquat University of Medical and Health Sciences, Jamshoro, PAK; 6 Urology and Obstetrics & Gynecology, California Institute of Behavioral Neurosciences & Psychology, Fairfield, USA; 7 General Surgery, Jawaharlal Institute of Postgraduate Medical Education and Research, Puducherry, IND; 8 Neurology, California Institute of Behavioral Neurosciences & Psychology, Fairfield, USA; 9 Internal Medicine, California Institute of Behavioral Neurosciences & Psychology, Fairfield, USA; 10 Obstetrics & Gynecology, California Institute of Behavioral Neurosciences & Psychology, Fairfield, USA; 11 Medicine, California Institute of Behavioral Neurosciences & Psychology, Fairfield, USA; 12 Medicine, Beni Suef University Faculty of Medicine, Beni Suef, EGY; 13 Surgery, Halifax Health Medical Center, Daytona Beach, USA; 14 Surgery, California Institute of Behavioral Neurosciences & Psychology, Fairfield, USA; 15 Surgery, Dr.Pinnamaneni Siddhartha Institute of Medical Sciences and Research Foundation, Gannavaram, IND; 16 Psychiatry and Behavioral Sciences, California Institute of Behavioral Neurosciences & Psychology, Fairfield, USA; 17 Division of Research & Academic Affairs, Larkin Community Hospital, South Miami, USA

**Keywords:** oncology, gut microbiota, dysbiosis, estrobolome, breast cancer, intestinal microbiome

## Abstract

Breast cancer is the most common cause of cancer-related deaths in women. Breast cancer is still a major cause of morbidity and mortality among women despite all the available diagnostic and treatment modalities. The gut microbiota has drawn keen interest as an additional environmental risk factor in breast cancer, especially in sporadic cases. This article explores factors that disrupt the normal gut microbial composition and the role of gut microbial dysbiosis in the development of breast cancer. We finalized 40 relevant articles after searching Pubmed and Google Scholar using regular keywords and the Medical Subject Headings (MeSH) strategy. Gut microbiota dysbiosis has been shown to play a role in the development of breast cancer via estrogen-dependent mechanisms and non-estrogen-dependent mechanisms involving the production of microbial-derived metabolites, immune regulation, and effects on DNA. The gut microbiota influence estrogen metabolism hence estrogen levels. The metabolites that have demonstrated anticancer properties include lithocholic acid, butyrate, and cadaverine. New approaches targeting the gut microbiota have come up and may yield new advances in the prevention, diagnosis, and treatment of breast cancer. They include the use of prebiotics, probiotics, and hormone supplements to restore normobiosis in the prevention and treatment of breast cancer.

## Introduction and background

The most common cancer in women, excluding skin cancers, is breast cancer. In 2020, female breast cancer was found to be the cancer with the highest incidence, with an estimated 2.3 million cases (11.7% of total cancer cases) surpassing lung cancer (11.4% of total cancer cases) for the first time [1]. It is currently the most frequent cause of cancer death in women worldwide, with about 685,000 estimated deaths in 2020 [1]. About one in eight women in the United States (US) will develop breast cancer in their lifetime [2]. The predicted worldwide incidence of female breast cancer by 2050 is estimated to be 3.2 million new cases per year [2]. These alarming numbers reflect the need for strict screening measures, prevention and treatment strategies, and further studies to understand all possible factors influencing the development of breast cancer. Incidence rates of breast cancer have been rising by about 0.5% per year, partially due to continued decline in the fertility rate and increased body weight [3].

Breast cancer risk factors have been widely known and discussed over the years [4]. A small subgroup (5-10%) of breast cancer cases have been linked to inherited gene mutations [5]. The concept of hormone-driven breast cancer is known, and appropriate treatment modalities are directed towards hormone receptor-positive cancers. Increased breast density on mammography and first-degree relatives with breast cancer were both associated with at least a two-fold increase in breast cancer risk in women aged 40-49 years [6]. Unfortunately, the etiology of sporadic breast cancer cases is not well known. Therefore, it is important to investigate factors involved in the development of sporadic cases, in order to develop appropriate prevention strategies. Microbiota refers to the entirety of microbes, both commensals and potentially pathogenic microbes (bacteria, archaea, fungi, viruses, and protozoa), in a particular habitat [7,8]. In this article, the word microbiota has been used primarily to refer to bacteria. Microbiome refers to the collection of genomes of microbiota and is commonly used to describe the entity of microbial traits or functions encoded by the microbiota [7]. The terms 'microbiota' and 'microbiome' are now often used synonymously [7,8].

Dysbiosis refers to an abnormal composition of the microbiome in the entire body or a body compartment [8]. There is evident variability of the microbiome between organs and from one individual to the other, as depicted in the Human Microbiome Project (2008 to 2013), making it a potential determinant of disease. The composition of the human microbiota has also been implicated in the development and aggressiveness of cancers [8]. The largest proportion of the human microbiota resides in the gut. Bacterial communities within a host could be one additional environmental factor related to breast cancer that has only been recently considered in sporadic breast cancers of unknown etiology [8]. Recently, efforts have been made towards fully characterizing the microbiota in different body parts under different health conditions, including breast cancer. Bacterial microorganisms interact with the host metabolic processes and can impact homeostasis. The most relevant strategies now used in microbiota research include 16S rRNA gene next-generation sequencing (NGS), a targeted approach, and a large-scale metagenomics approach, also known as shotgun sequencing [8]. Both quantitative and qualitative differences between gut microbiota in healthy people and people with breast cancer have been explored in a few studies, but a conclusive disease-causing microbial profile has not been determined.

This article aims to elaborate on the role of the disruption of the gut microbiome (dysbiosis) in breast cancer development via estrogen-dependent and non-estrogen-dependent mechanisms [9]. We discuss the causes of microbial dysbiosis and how its prevention or correction can reduce the risk of sporadic breast cancers. Another aim of this article is to shed some light on the new possibilities of involving interventions targeting the intestinal microbiome in diagnosing and treating breast cancer. This article also explores the relevance of the identification of the gut microbiome and its metabolites in stratifying patients for the risk of breast cancer. Potential treatment strategies for breast cancer targeting the gut microbiome, such as prebiotics, probiotics, and dietary interventions, are still in their infancy and are explored further in this review.

## Review

Method

We performed a search on PubMed and Google Scholar using regular keywords and using the MeSH strategy; 'Breast microbiome' OR 'microbiota' AND 'gastrointestinal' OR 'gut' OR 'fecal microbiome' AND 'dysbiosis' AND 'breast cancer' (Table 1 ). We included articles with age groups above 19 years, studies with female subjects, and the English language. We excluded articles and trials not related to gut microbial dysbiosis and the role of gut microbiota in breast cancer in general.

**Table 1:** Table showing keywords and search strategy used in the database search

Search strategy	Database used	Number of articles before customization and screening	Number of articles after customization and screening
Breast Microbiome OR Microbiota	Pubmed	651	33
Gastrointestinal OR Gut OR Microbiome OR Microbiota	Pubmed	1572	19
Fecal microbiome OR microbiota in Breast cancer	Pubmed	155	45
Intestinal Dysbiosis in Breast cancer	Pubmed	6	6.
Dysbiosis in Breast cancer	Pubmed	27	22
Breast Cancer	Pubmed	122,048	_
Breast microbiome OR microbiota AND Gastrointestinal OR Gut OR Fecal Microbiome OR Microbiota AND Dysbiosis AND Breast Cancer (MeSH search)	Pubmed	44	44
Gut microbiota dysbiosis in Breast Cancer.	Google Scholar	5	5

Results

We screened the articles by title and by the content of the abstracts and full texts. The total number of articles after the initial screening process was 174. Duplicates were removed leaving 87 articles. After further screening by content, the final number of articles used for this paper was 43, comprising two clinical trials, one systematic review and meta-analysis, 17 review articles, 19 observational studies, three animal studies, and an in vitro study. 

Discussion

Factors influencing gut microbiota composition

**Figure 1:**
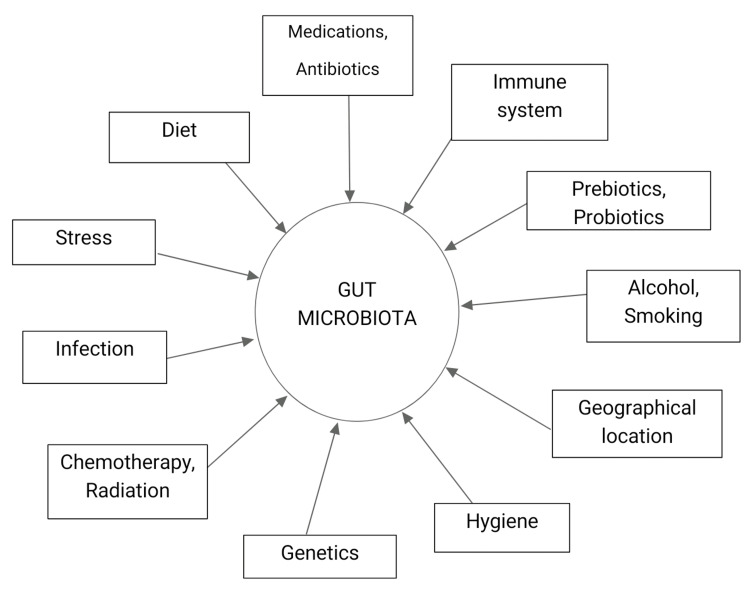
Factors that influence gut microbiota composition causing dysbiosis.

The main phyla of the gut microbiota are Firmicutes, Bacteriodetes, Actinobacteria, Proteobacteria, Fusobacteria, Verrucomicrobia, Tenericutes, and Lentisphaerae [10]. The main genera are *Bacteroides, Clostridium, Faecalibacterium, Eubacterium, Ruminococcus, Peptococcus, Peptostreptococcus, Lactobacillus, Streptococcus, Streptomyces, *and *Bifidobacterium* [10]. The gut microbiota helps in digestion, metabolism, and immune response, giving rise to a symbiotic relationship between the host's gut and the microbiota called normobiosis, which maintains homeostasis [10]. Dysbiosis disrupts the normal gut-microbiota interaction [10]. It occurs when harmful bacteria outcompete beneficial bacteria (commensals) and/or a reduction in alpha diversity which can lead to disease [10]. Factors influencing the gut microbiota composition include host factors like age, ethnicity, hormonal levels, and environmental factors such as diet, prebiotics, probiotics, stress, hygiene, alcohol, smoking, antibiotic use, chemotherapy, and radiation (Figure 1) [11]. The most explored factors are diet, antibiotics, prebiotics, and probiotics and they are briefly described below.

Diet

Distinct patterns of intestinal microbiota composition are associated with habitual consumption of animal fats, dietary fiber, and vegetables [12]. High-fiber diet influences gut microbiota composition and lower gut bacterial beta-glucuronidase activity [10]. This reduces deconjugation and reabsorption of estrogens, increasing the fecal excretion of estrogen compounds, leading to a reduction in estrogen levels. Vegetarian women had an increased fecal output leading to a three times increase in fecal excretion of estrogen, reduced fecal glucuronidase activity (p<05), and a resultant 15-18 % decrease in plasma estrogen levels when compared with non-vegetarian women [13,14]. Immigrant women on a low-fat diet (20%-25% of calories) had lower systemic estrogen levels than American women eating a high-fat diet (40% of calories) by 30% [13]. Further correlation in the study, showed that plasma estrogen levels were directly associated with fat and inversely associated with fiber [13]. The Western-type diet increases sex hormone levels and decreases the sex hormone-binding globulin increasing steroid hormone availability for tissues like the breast, giving a sex hormone pattern that is commonly seen in breast cancer [15]. Newman et al. reviewed the antineoplastic molecular mechanisms of the Mediterranean diet on the gut microbiome and concluded that the Mediterranean diet could be beneficial in the prevention and treatment of breast cancer [12]. An association between diet and estrogen metabolism has been noted. Dietary fiber can induce bacterial enzyme selectivity by influencing the microbial composition, although other factors may contribute [10]. It also influences bile acid metabolism by interrupting the enterohepatic circulation [10]. Thus, the diet directly affects the gut microbiota composition, estrogen levels, and production of microbial-derived metabolites, which may impact the risk of breast cancer.

Antibiotics, Prebiotics, and Probiotics

Antibiotics usage can alter the gut microbiota composition depending on the class of antibiotic, dose, period of exposure, and target bacteria from the mode of action [10]. Some studies showed that antibiotic consumption (especially prolonged use) was associated with an increased risk of breast cancer development and recurrence, possibly by reducing the diversity of gut microbiota, with one of the studies, describing only a weak correlation [16-18]. In contrast, two studies by Sorensen et al. and Garcia Rodriguez et al. found no increase in the risk of breast cancer with antibiotic use [19,20]. Given the inconclusive evidence, a cause-and-effect relationship cannot be established, and some uncontrolled confounding factors may be involved, such as hormone-related diseases that the antibiotics were used for, like acne [18]. Despite the conflicting studies, there is still a cause for prudent use of antibiotics, especially for long-term purposes. Further studies are needed to establish whether an association exists between antibiotic use and breast cancer and this would strengthen the hypothesized relationship between gut microbiota perturbation and breast cancer.

On the other hand, prebiotics and probiotics help maintain proper microbial composition and restore normal balance. Prebiotics such as enterolactone is typically indigestible fiber compounds that enhance the growth and activity of beneficial gut microorganisms. Some, such as phytoestrogens, work as antioxidants causing downregulation of cyclooxygenase-2 (COX-2) mediated inflammation and are shown to be beneficial in cancer prevention [10]. Probiotics are living microorganisms that, when administered in adequate amounts, confer a health benefit to the host [10]. Consumption of probiotics containing live bacteria such as *Lactobacillus *spp. causes changes in the intestinal microbiome composition and has been shown to decrease the activity of fecal beta-glucuronidase [21,22]. This reflects a decrease in estrogen levels and hence a decrease in the risk of breast cancer.

The crosstalk between the gut microbiota and breast cancer

Breast cancer development is influenced by estrogen-dependent and non-estrogen-dependent functions of the gastrointestinal microbiome involving the production of bioactive metabolites [10,23]. Intestinal microbiota dysbiosis has been linked to breast cancer because certain gut bacteria alter the production of the beneficial anticancer metabolites and disrupt estrogen metabolism in the gut.

Estrogen Dependent Effects: Gut Microbiota-Estrobolome-Breast Cancer Connection

**Figure 2:**
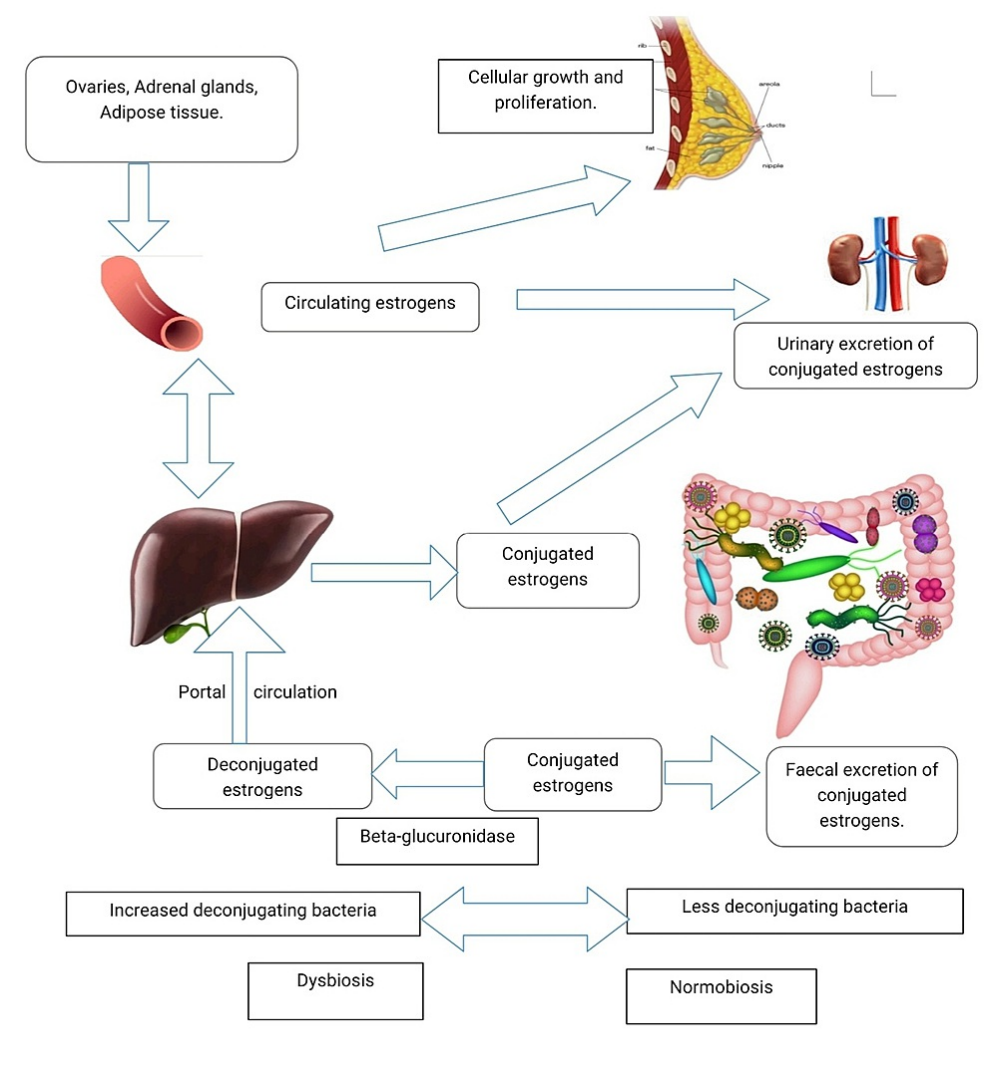
The effect of the gut microbiota on the metabolism and enterohepatic cycling of estrogen Adapted from: Kwa M., et al., Natl Cancer Inst, 2016 [11].

The risk of estrogen-driven breast cancer in postmenopausal women is associated with the concentration of circulating estrogens and duration of exposure to estrogen [24]. The estrobolome refers to the aggregate of intestinal bacterial genes whose products metabolize estrogen, hence may influence the risk of estrogen receptor-positive breast cancer in postmenopausal women [25]. Estrogens are conjugated in the liver and released into bile, which then enters the intestine. Some intestinal bacteria secrete beta-glucuronidase, which deconjugates estrogens that were marked for fecal excretion allowing binding to their receptors [26]. Ervin et al. showed that human gut microbial beta-glucuronidase enzymes could reactivate two estrogen glucuronides, estrone-3-glucuronide and estradiol-17-glucuronide, to estrone and estradiol [27]. However, inhibition of this estrogen reactivation did not prevent tumor development in the mouse model of breast cancer, meaning there are many other processes involved [27]. The free estrogens are reabsorbed via the enterohepatic circulation and reach distant tissues like the breast (Figure 2) [8]. In addition, gut microbes break down indigestible dietary polyphenols to biologically active estrogen-like metabolites with varying estrogenic potency. Gut bacteria also induce the synthesis of estrogen inducible growth factors such as estromedin, which can be involved in carcinogenesis [8,28]. Some commensals have the capability via sulfatase to convert inactive steroids into active estrogen [24]. Activation of estrogen receptors in the breast increases the number of cells entering the G0 and G1 phases, stimulating cell proliferation, which is a common finding in breast cancer [29]. Activation of the estrogen receptors in mitochondria induces mitochondrial oxidation, resulting in increased mitochondrial production of free radicals, promoting breast cancer development [23]. 

The proportion of beta-glucuronidase-producing gut bacteria influences the levels of estrogens and their potential breast cancer-associated effects. Disproportionally high levels of such bacteria may be present during dysbiosis. Bacterial beta-glucuronidases are encoded by *GUS* genes found in most gut bacteria, most commonly Firmicutes, and *BG* genes present in Firmicutes and Bacteroidetes [23]. Beta-glucuronidase-producing bacteria include *Bacteroides, Bifidobacterium, Clostridium, Citrobacter, Escherichia, Faecalibacterium, *and *Propionibacterium*, to name a few [30,31]. Therefore, beta-glucuronidase deconjugation activity is dependent on gut microbial composition and diet [26]. Bard et al. found that absolute numbers of *Bifidobacterium* and *Blautia* varied significantly according to the clinical stages of breast cancer patients, and other bacterial counts varied with BMI [31].

The effect of the gut microbiome diversity on the levels of estrogens and their metabolites has been explored in various studies. Fuhrman et al. found that healthy postmenopausal women with increased gut microbiome diversity and an abundance of four Clostridia taxa had an increased urinary ratio of hydroxylated estrogen metabolites to parent estrogens, which is related to the etiology of breast cancer [32]. Similarly, Flores et al. showed that total urinary estrogen levels were directly associated with fecal microbiome richness, alpha diversity (R≥0.50, P≤0.003), and Clostridia taxa in healthy postmenopausal women and men but not in premenopausal women [33]. Fecal beta-glucuronidase activity correlated significantly with estrone levels and inversely with total estrogens in older adults [33]. This suggests that the influence of gut microbiota on estrogen-driven diseases is limited to older women and men but is absent in premenopausal women. Likewise, a metagenomic analysis demonstrated differences in the functions and composition of gut microbiota amongst the subjects [34]. Postmenopausal breast cancer patients had a different relative abundance of gut microbiota and higher microbial diversity compared to controls [34]. However, no remarkable discrepancy was found between premenopausal cases and controls [34]. In contrast, Goedert et al. concluded that postmenopausal women with breast cancer had altered gut microbiota composition but with estrogen-independent low alpha diversity (p=0.04) [35]. The contrast in these findings on levels of gut microbiota diversity and breast cancer could be because the microbiota can also be affected by the stage and outcomes of the disease [10]. In that regard, Miko et al. observed that the most dramatic decrease in microbial diversity was observed in the early stages of breast cancer [36]. These studies imply that variations in gut microbial diversity amongst women result in differences in estrogen levels, influencing breast cancer development. Further investigation of the correlation between specific microbiota profiles, for example, Clostridia taxa and estrogen levels, can identify potential targets for intervention [33]. As demonstrated in these studies, the gut microbiome is an important determinant of estrogen metabolism in postmenopausal women (Table 2).

**Table 2:** Observational studies showing the association between gut microbiota diversity, estrogen levels, and breast cancer.

Author	Year	Study type	No.	Age	Purpose	Results	Conclusion
Bard JM et al. [31]	2015	Observational study: Cross-sectional study	32	-	To assess the relationship between microbiota composition and clinical stages of breast cancer.	The absolute number of gut bacteria differed according to clinical grades and BMI. Patients with grade 3 breast cancer had relatively more absolute numbers of *Blautia spp.*	Gut microbiota composition differs according to clinical grades of breast cancer and BMI.
Fuhrman BJ et al. [32]	2014	Observational: Cross-sectional study.	60	55-69 years	To investigate whether urinary estrogens and estrogen metabolite levels are associated with the diversity and composition of the fecal microbiome.	The ratio of metabolites to parents was directly associated with phylogenetic diversity (R = 0.35, P = .01) and relative abundances of the order Clostridiales (R = 0.32, P = .02) and the genus *Bacteroides *(R = −0.30, P = .03).	Women with higher gut microbiome diversity had an increased urinary ratio of hydroxylated estrogen metabolites to parent estrogens.
Flores et al. [33]	2012	Observational study: Cross-sectional study	51	25 men, seven postmenopausal women, and 19 premenopausal women	To identify members and functions of the intestinal microbiota that influence estrogen levels via enterohepatic recirculation.	Urinary estrogen levels in men and postmenopausal women correlated directly with fecal microbiome richness and alpha diversity (R≥0.50, P≤0.003) and fecal Clostridia taxa. In addition, fecal β-glucuronidase correlated proportionally with estrone and inversely with total fecal estrogens (R≤-0.47, P≤0.01). However, in premenopausal women, these associations were absent (P≥0.6).	Levels of non-ovarian estrogens are associated with gut microbial richness and diversity. This likely affects the risk for estrogen-related conditions in older adults but not in premenopausal women.
Zhu et al. [34]	2018	Observational: Case-control study	133	Premenopausal cases and controls, postmenopausal cases and postmenopausal controls	To evaluate the composition and functional capacity of gut microbiota of breast cancer patients compared to controls.	Relative abundance of gut microbiota species differed significantly, and microbial diversity was higher in postmenopausal breast cancer cases and controls but did not differ between premenopausal cases and controls. The gut metagenomes of postmenopausal breast cancer patients had many genes encoding lipopolysaccharide biosynthesis, iron complex transport system, secretion system, and beta-oxidation.	There were notable differences in the gut microbial composition and activity between postmenopausal breast cancer patients and healthy controls. The gut microbiota also influences host immunity and metabolic balance.
Goedert et al. [35]	2015	Observational: Case-control Pilot study	96	Postmenopausal women	To seek differences between the gut microbiota in 48 postmenopausal breast cancer cases and 48 controls.	Cases had altered microbiota composition (β-diversity, P = .006) and lower α-diversity (P = .004) compared to controls.	Postmenopausal women with breast cancer have altered composition and estrogen-independent low diversity of their gut microbiota.

Obesity: Gut microbial dysbiosis can lead to obesity [37]. The concept of microbe-induced obesity was proposed as a condition where increased adiposity occurs due to primary disruption of the microbiota in early life by various factors such as antibiotics [37]. Obesity is a known breast cancer risk factor mostly via its effect on estrogen levels. Increased adiposity increases the risk of breast cancer through peripheral aromatization of androgens and suppression of production of hepatic hormone-binding proteins resulting in elevated free and total estrogens [11]. A relationship between cancer risk and adult weight gain was described where each 5-kilogram increase in weight was associated with an additional 11% increase in the risk of postmenopausal breast cancer [37]. Therefore, modulation of the gut microbiota to restore normobiosis can be a targeted approach against obesity and its associated breast cancer risk. Administration of soy isoflavones to postmenopausal women has been demonstrated to suppress Clostridiaceae, a family of Clostridiae that has been linked to obesity [10]. This would reduce the risk of breast cancer associated with obesity. 

*Non-Estrogen-Dependent Effects of the Gut Microbiota*

Gut bacteria can play a protective role by producing cancer-protective metabolites. They also have an impact on immune regulation and DNA integrity.

Metabolism of bile acids: Bile acids found in the breast tissue originally come from the gut [23]. Lithocholic acid (LCA) is a bile acid produced only by intestinal bacteria such as *Clostridia *spp. from primary bile acids [23]. It has been found to exert antitumor effects with a 10-20% decrease in breast cancer cell proliferation and inhibition of epithelial to mesenchymal cell transition [23,38]. It has antitumor immunity in murine models and in vitro, acting via the TGR5 receptor [23,38]. It also increases p53 expression, hence reducing cell death, and inhibits vascular endothelial growth factor (VEGF) production [23-24,38]. LCA causes changes in cellular metabolism by inducing glycolysis, the tricarboxylic acid (TCA) cycle, and mitochondrial oxidative phosphorylation in breast cancer cells that depend on Warburg metabolism [24,36]. Lithocholic acid (LCA) levels and levels of the LCA-producing enzyme gene were found to be low in fecal DNA of cases with early breast cancer relative to controls indicating reduced biosynthesis by gut microbiota in early breast cancer [36]. This shows the correlation between the composition of gut microbiota, lithocholic acid production, and its antiproliferative effects on breast cancer, which presents a potential target for intervention.

Production of short-chain fatty acids: Short-chain fatty acids (SCFAs), mainly butyrate, propionate, acetate, and lactate, are generated from the fermentation of indigestible carbohydrates by the intestinal microbiome and a small proportion by degradation of branched-chain amino acids [23]. The most protective SCFA is butyrate, which has the anticancer activity of inducing cancer cell apoptosis via mitochondrial reactive oxygen species (ROS) generation, anti-inflammatory effects, and inhibition of histone deacetylation via the Warburg effect [10,23,38]. Butyrate also suppresses angiogenesis, inhibiting tumor formation [38]. Its concentration in the intestine is dependent on diet and gut microbiota composition [38]. A high-fiber diet promotes butyrogenesis and proliferation of butyrate-producing microbes and, hence, is considered cancer-protective. Important butyrate-producing microbes include *Faecalibacterium prausnitzii, Roseburia intestinalis, *and *Eubacterium rectale* [38]. Reduction in the proportion of butyrate-producing bacteria could increase tumorigenesis and inflammation [10]. Therefore, butyrate is a potential anticancer metabolite whose production can be facilitated by dietary changes that modulate the gut microbiome. This can be applied in breast cancer prevention and treatment adjunctively.

Amino acid degradation: Metabolism of dietary amino acids produces biogenic amines with various functions. Cadaverine, a biogenic amine, is preferentially synthesized by bacterial enzymes from the decarboxylation of lysine [23]. Bacteria with the ability to produce cadaverine include members of the genera *Enterococcus, Enterobacter, Escherichia, Proteus, Streptococci, *and *Shigella* [24,38]. Cadaverine supplementation in vitro was found to inhibit breast cancer cells growth by inhibiting cellular migration, invasion, metastasis, and suppressing epithelial to mesenchymal transition [23,38]. It acts via trace amino acid receptors (TAAR) on target cells [24,38]. Its levels were found to be reduced in patients with early-stage breast cancer, possibly due to decreased microbial production demonstrating its role in the reduction of breast cancer risk and progression [39].

DNA damage: Intestinal microbes can trigger malignant transformation by disrupting genomic stability, causing resistance to cell death, and altering cell proliferation [26]. In addition, gut microbiota can cause damage to the host DNA by causing carcinogenic mutations and by releasing toxins into host cells, for example, from *Bacillus fragilis*, which can cause carcinogenic cell responses [26]. Increased reactive oxygen species production induced by some bacteria also damages DNA [26].

Immune regulation: Microbes can facilitate malignancy by stimulation of an unregulated inflammatory immune response [26]. Dysbiosis results in shifts in the bacterial metabolites toward an inflammatory state that favors and propagates carcinogenesis [23]. Gut bacteria were found to require neutrophils to promote tumorigenesis in distant sites such as the mammary gland in mice, demonstrating the involvement of immune cells in tumor formation [40]. An intact intestinal barrier is an essential component of the immunity that prevents translocation of gut bacteria to distant sites such as the breast and the release of lipopolysaccharide (LPS) from gut bacteria into the blood [38]. A healthy gut barrier involves the maintenance of tight junctions between epithelial cells by proteins such as claudins and the presence of a thick mucus lining maintained by some bacteria [38]. A state of dysbiosis compromises the cellular adhesion proteins and reduces the bacterial species that maintain the thick mucus lining leading to increased barrier permeability, also referred to as a 'leaky gut' [38]. An imbalance between regulatory T cells and inflammation-associated Th17 cells (increased) also occurs, inciting both local and systemic inflammatory responses [38]. Increased circulating LPS has been associated with breast cancer metastasis via activation of monocyte-activated endothelial adhesion of circulating cancer cells [38]. On the other hand, *Faecalibacterium prausnitzii* was found to suppress the growth of breast cancer cells via its inhibitory action on the IL6/STAT3 pathway showing a potential avenue for intervention that should be explored further [41].

Application to diagnosis, prevention, and treatment of breast cancer

The intestinal microbiome can be useful in the diagnosis of breast cancer. The microbiota of breast cancer patients needs to be profiled to allow us to conclusively determine if a causative association exists between gut microbiota and breast cancer. This would identify the gut microbiota as an additional environmental risk factor and a potential prognostic modulator of the disease [42]. An ongoing case-control clinical trial aims to demonstrate the association between breast cancer and the mammary and intestinal microbiota characteristics by undertaking simultaneous analysis of both [42]. It also attempts to link the microbiota with endocrine disruption and the risk of breast cancer [42]. Investigating variations in composition and functionality of the estrobolome in healthy individuals relative to estrogen-driven breast cancer patients can lead to the development of microbiome-based biomarkers and future targeted interventions to reduce the risk of breast cancer [11]. Correlations to specific bacterial taxa associated with breast cancer, such as Clostridia should be carefully explored as part of potential cancer detection strategies [33]. Ma et al. found that *Faecalibacterium *abundance was reduced in breast cancer patients and had a negative correlation with phosphorocholine [36]. This implies that combining the detection of flora bacteria such as *Faecalibacterium* and flora metabolites such as phosphorolcholine could be useful in breast cancer detection [36].

Disruption of the gut microbiome results in variations in the microbiome-derived cancer-protective metabolites, promoting carcinogenesis in distant organs, in this case, the breast. Thus, strategies to promote a gut microbiota profile that enables the production of cancer-protective bacterial metabolites via targeted dietary or supplementary interventions in women with breast cancer could improve their health outcomes. Although precise mechanisms remain undetermined, this is an up-and-coming area to explore. Interventional clinical studies should be designed with the aim of restoring the gut dysbiosis found in breast cancer patients toward a composition similar to that of healthy women [42]. Prebiotics and probiotics can be considered for use to prevent or arrest breast cancer development by restoring normobiosis [10]. Their active compounds can be purified and used as adjunctive therapy. Soy isoflavones such as daidzein are prebiotics from a class of phytoestrogens that are metabolized by bacteria to a biologically active metabolite called equol that has been shown to have defensive activity against breast cancer, among other diseases [43]. However, only about 30-50% of people can convert isoflavones to equol, probably due to variations in microbiota composition. In this regard, investigation needs to be done on improving the production of equol, perhaps by administering daidzein with probiotic bacteria as a potential preventative strategy against breast cancer in menopausal women [43]. Dietary fiber is a well-known form of a prebiotic that is fermented by gut bacteria producing cancer-protective metabolites such as butyrate [36]. There is an ongoing interventional clinical trial whose objective is to investigate the effect of a novel probiotic on the breast and gut microbiome in breast cancer as well as on the quality of life of breast cancer patients [44]. Herbal supplements such as American ginseng can produce cancer-protective metabolites and regulate intestinal microbiota composition to prevent tumorigenesis [10]. The spores of *Ganoderma lucidum *have generated interest due to their anti-inflammatory and anticancer properties that result from restoring exhausted T cells by increasing cytotoxic T cell population and downregulating the suppressive co-inhibitors; programmed cell death protein (Pd-1) and cytotoxic T lymphocyte antigen-4 (CTLA-4) [45]. *​Ganoderma lucidum *spores could also be used to modulate the intestinal microbiota and restore normobiosis [45]. Conclusive clinical trials need to be done to demonstrate the benefits of incorporating these substances to prevent and treat breast cancer. 

## Conclusions

The burden of breast cancer still looms over our heads, and we ought to stay ahead of the curve. Gut microbial dysbiosis seemingly has a role to play in the development of some cases of breast cancer and has untapped life-saving potential as a target for intervention. Several factors disrupt normobiosis in the gut and homeostasis, favoring the development of disease. The gut bacteria influence estrogen metabolism and adiposity, which are both associated with breast cancer. The microbiota are also involved in producing cancer-protective metabolites such as butyrate, cadaverine, and lithocholic acid. Immune interactions with gut microbiota and microbial effects on host DNA also play a role in the development of breast cancer and are potential targets of treatment. The evidence of all these correlations marks the gut microbiome as a hub for further exploration into novel approaches in diagnosis and treatment. Once well characterized, the gut microbiota profile and its metabolites may serve as biomarkers for breast cancer. Prebiotics, probiotics, and supplements should be explored for use in the prevention and adjunctive treatment of breast cancer by reversal of gut microbial dysbiosis. A few animal and in-vitro studies were incorporated in this review. We were limited by the paucity of human interventional clinical trials to validate the suggested treatment approaches and incomplete profiling of the intestinal microbiome of breast cancer patients making it difficult to ascertain a causal relationship between gut microbiota dysbiosis and breast cancer. Interventional clinical trials, where safe, are required to substantiate the use of the prevention and treatment strategies mentioned above. Large-scale profiling of the gut microbiome and the associated metabolome of breast cancer patients relative to that of healthy individuals will be beneficial to allow the determination of new biomarkers for the disease. 
